# Data on kilometer scale production of stretchable conductive multifilaments enables knitting wearable strain sensing textiles

**DOI:** 10.1016/j.dib.2018.04.090

**Published:** 2018-05-01

**Authors:** Shayan Seyedin, Sepehr Moradi, Charanpreet Singh, Joselito M. Razal

**Affiliations:** Deakin University, Institute for Frontier Materials, Geelong, VIC 3220, Australia

## Abstract

This data article contains analyzed data for the article “Continuous Production of Stretchable Conductive Multifilaments in Kilometer Scale Enables Facile Knitting of Wearable Strain Sensing Textiles” (Seyedin et al., 2018) [1]. Details of wet-spinning conditions to achieve scaled-up production of stretchable and conducting polyurethane/poly(3,4-ethylenedioxythiophene):poly(styrenesulfonate) (PU/PEDOT:PSS) multifilaments are provided. The stress-strain curves for tensile and stretch-relaxation tests on the multifilament and different knitted textile structures (plain-knit, co-knit, co-knit-alternate, co-knit with conductive stitch, and plain with non-conductive stitch) are presented. It is shown that the PU/PEDOT:PSS multifilaments can also be knitted into fabrics that when worn on various body parts, such as knee, elbow, and finger, can monitor their various movements.

**Specifications table**

**Table:** 

Subject area	Materials Engineering, Chemistry
More specific subject area	*Functional fiber fabrication, wearable strain sensors*
Type of data	*Figures, table, electron microscopy image, photographs, videos*
How data was acquired	*Custom-built wet-spinning machine (Dissol Pty. Ltd.), tensile testing instrument (Instron 5967), field emission scanning electron microscope (SEM, JEOL JSM-7800F)*
Data format	*Analyzed*
Experimental factors	*Poly(3,4-ethylenedioxythiophene):poly(styrenesulfonate) (PEDOT:PSS) pellets (Agfa Orgacon*^*TM*^*Dry) were dispersed in dimethyl sulfoxide (DMSO) using a homogenizer (IKA T25 Digital, ULTRA-TURRAX) at* 25,000* *rpm *for about* 1* *h*. Polyurethane (PU, AdvanSource Biomaterials Chronoflex*^*®*^*C* 80* *A*) solutions in DMSO or dimethyl formamide (DMF) were separately prepared and mixed with the PEDOT:PSS solution to produce spinning formulations.*
Experimental features	*The PU/PEDOT:PSS spinning formulation was injected into a coagulation bath through a 100-hole spinneret using a metering pump at a controlled flow rate of ~1 *mL* *min^−1^*. The fibers were then passed continuously through several wash baths and a non-contact heating column at 80 °C for drying and were finally collected onto a spool.*
Data source location	*Institute for Frontier Materials, Deakin University, Geelong, VIC 3220, Australia*
Data accessibility	*Data are provided with this article*

**Value of the data**

• The data presented here shows how we can solve some of the challenges in transitioning from lab-scale wet-spinning of functional monofilaments to scaled-up fabrication of multifilaments which are suitable for textile processing.
• The data in this article provide details of the experimental conditions to achieve stretchable and conductive PU/PEDOT:PSS multifilaments.
• The tensile and stretch-relaxation data of multifilament and textile prototypes may be used to elucidate the effects of structure on strain sensing behavior of the knitted textiles.
• The data explain in details how to use the strain sensing textile prototypes for wireless monitoring of movements of various body parts.

## 1 Data

This Data in Brief article describes the fabrication of stretchable and conductive PU/PEDOT:PSS multifilaments via a scaled-up wet-spinning process and the mechanical and strain sensing properties of the multifilaments when used individually and within various knitted textile structures as shown in Ref. [1]. We have previously shown that PU/PEDOT:PSS monofilaments can be spun continuously using a laboratory bench-scale wet-spinning set-up [2], [3]. The current technology of novel fibers fabrication only exists in bench-scale. This is because fiber spinning is a complex process that largely depends on the spinning formulations and coagulation bath chemistries and a number of process parameters. In order to achieve scaled-up wet-spinning, it is critical to have high-quality, highly exfoliated, aggregate-free, and uniform composite formulations in solutions. For instance, the presence of large particles can result in the blockage of the nozzle of the spinneret (needle) or can cause fibers to break frequently during spinning. Inefficient solidification caused by inappropriate coagulating composition can result in the formation of droplets or broken fibers instead of a continuous filament. Hence, an in-depth study is required to carefully control and to optimize the spinning parameters in order to achieve continuous PU/PEDOT:PSS composite multifilaments at an scale appropriate for textile processing.

**Fig. 1:**
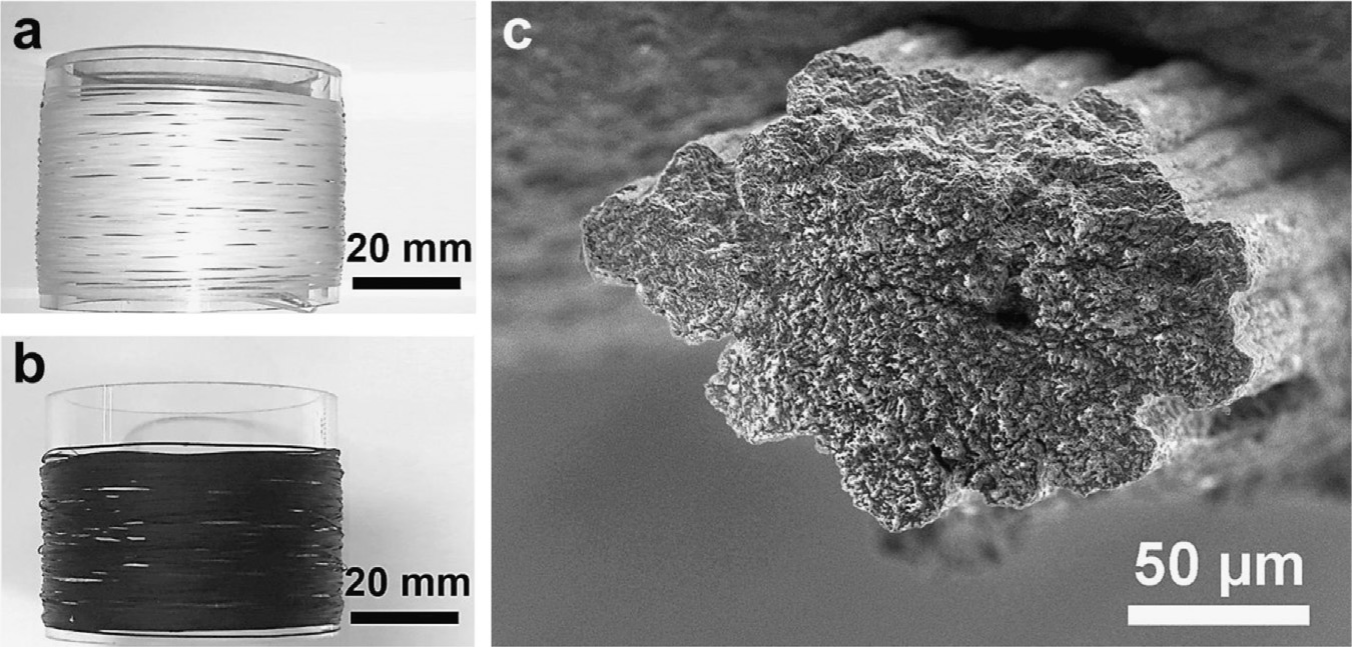
Digital photographs of (a) PU and (b) PU/PEDOT:PSS multifilaments produced using a lab-scale wet-spinning setup. (c) SEM image of PU/PEDOT:PSS fiber cross-section showing filament sticking when unsuitable spinning conditions are used.

Table S1 summarizes the experiments that were carried out in order to achieve suitable wet-spinning conditions for scaled-up fabrication of stretchable and conductive PU/PEDOT:PSS multifilaments. We started from a simple case of pure PU multifilament wet-spinning using a bench-scale setup. We prepared PU spinning solutions by dissolving the PU granules (200 mg mL^−1^) in several solvents including dimethyl sulfoxide (DMSO), dimethyl formamide (DMF), and DMSO/DMF (50/50 V/V). We used a 20-hole spinneret (nozzle internal diameter of 150 µm and L/D ratio of 3) for spinning PU multifilaments. We observed that when we used water as the coagulating solvent, sever filament sticking occurred and there was no clear boundary between the filaments. We then replaced water in the coagulation bath with isopropanol (IPA). While PU multifilaments were achieved from the DMF solution, wet-spinning of PU solutions in DMSO and DMSO/DMF using IPA in the coagulation bath led to droplets or short fiber fabrication. The low coagulation rate in these solvent/non-solvent systems could be the reason for the unsuccessful fiber spinning. We found that it was possible to achieve spinnability form all solutions using an IPA/water (50/50 V/V) coagulation bath with the presence of noticeable filament sticking from DMSO and DMSO/DMF formulations. Interestingly, further increasing the IPA content to IPA/water 90/10 V/V resulted in PU multifilaments with distinct separation of filaments from all formulations (Fig. 1a).

While IPA/water 90/10 V/V was suitable for PU multifilament spinning, we observed that this coagulation composition was inefficient for PU/PEDOT:PSS composite formulation (PEDOT:PSS loading ~9 wt%) in DMSO as it resulted in frequent breakage of the filaments within the coagulation bath. By slightly decreasing the IPA content of the coagulation bath to IPA/water 80/20 V/V, we achieved continuous spinnability of the PU/PEDOT:PSS formulation in DMSO, albeit with a noticeable filament sticking (Fig. 1c). By changing the spinning solvent to a mixture of DMSO and DMF (50/50 V/V), we were able to achieve PU/PEDOT:PSS multifilament with clearly separated filaments (Fig. 1b, see Ref. [1] for the scanning electron microscopy images). This PU/PEDOT:PSS formulation was made by first dispersing the PEDOT:PSS pellets in DMSO using a homogenizer and then mixing the dispersion with a separately prepared PU solution in DMF. In the next step, we used a custom-built scaled-up wet-spinning machine equipped with a 100-hole spinneret (nozzle diameter of 100 µm and L/D ratio of 1.5) and successfully produced more than 1 km multifilaments using the PU/PEDOT:PSS formulations in DSMO/DMF (50/50) solvent mixture and IPA/water 80/20 V/V coagulation bath (see Ref. [1] for the digital photograph of the spool). The PU/PEDOT:PSS multifilaments were continuously washed and dried by passing through a series of washing baths (ethanol) and a heating column (heated to 80 °C) and high-quality PU/PEDOT:PSS multifilaments with the PEDOT:PSS loading of up to ~15 wt% were collected on a spool. Video 1 shows a short clip of the PU/PEDOT:PSS multifilament wet-spinning process (see [1] for details of the wet-spinning parameters). Due to the presence of some filament breakage and spinneret blockage with the PU/PEDOT:PSS formulation containing ~15 wt% PEDOT:PSS, we used multifilaments with ~13 wt% PEDOT:PSS in our knitting experiments.

**Fig. 2:**
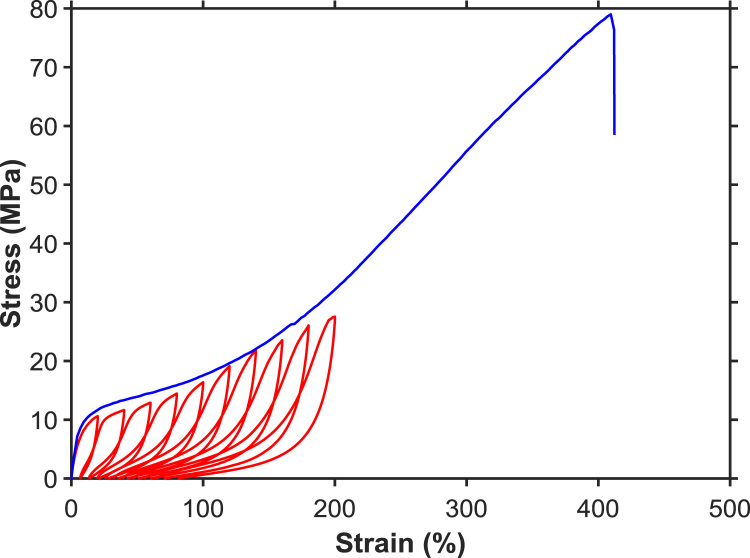
Representative stress–strain curves for tensile and cyclic stretch-relaxation tests on PU/PEDOT:PSS multifilaments.

**Fig. 3:**
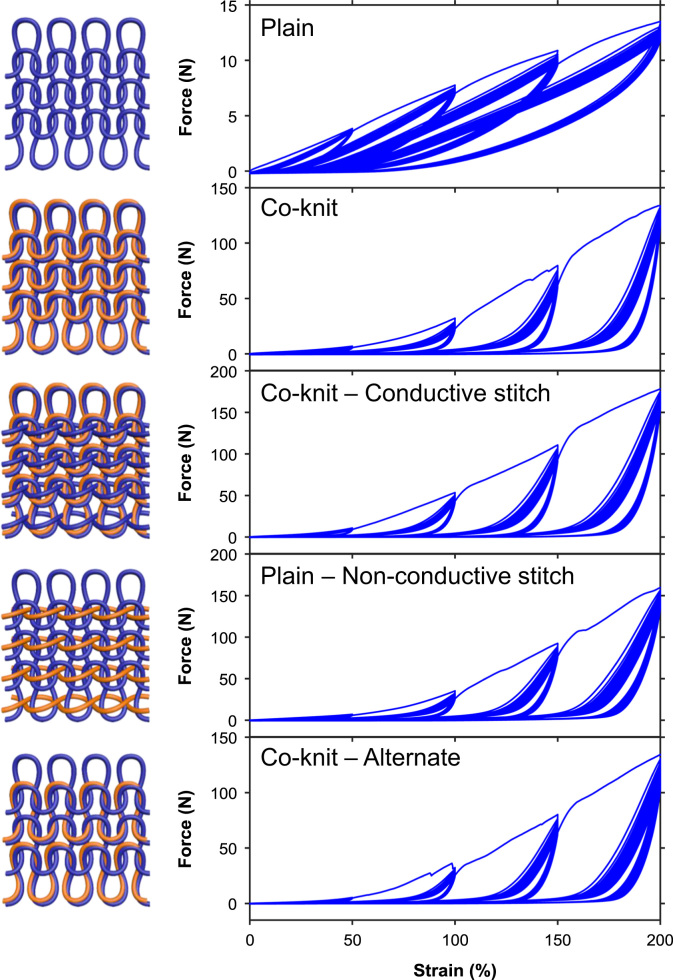
Representative stress–strain curves for cyclic stretch-relaxation tests on various knitted textiles.

**Fig. 4:**
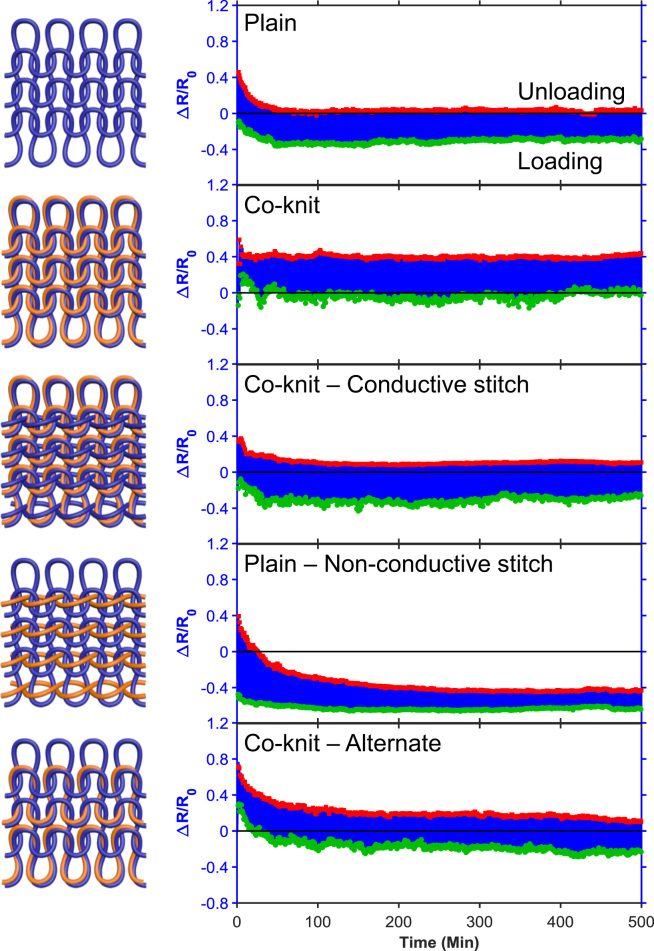
Long-term stability of various textile structure at 100% stretching evaluated using cyclic electromechanical tests.

Fig. 2 shows typical stress-strain curves of the PU/PEDOT:PSS multifilament (PEDOT:PSS loading ~13 wt%) measured from uniaxial tensile and stretch-relaxation tests. The PU/PEDOT:PSS multifilament showed a Young's modulus of ~142.8 MPa, tensile strength of ~76.3 MPa, breaking strain of ~414.8%, toughness of ~145.3 MJ m^−3^, and elastic recovery of ~70% at the applied strain of 100%. The excellent mechanical properties of the PU/PEDOT:PSS multifilaments enabled their knitting into various structures such as plain-knit, co-knit, co-knit-alternate, co-knit with conductive stitch, and plain with non-conductive stitch (see Ref. [1] for details of the knitted structures). The cyclic stretch-relaxation data (Fig. 3) shows that all knitted textiles could be stretched to 200% with no evidence of damage in the structure. It also shows very high recovery of the textiles when cyclically stretched to various strains. We observed that the force required to achieve a specific strain was noticeably different in various structures; the plain-knit required the lowest force for stretching and as the structure became denser through the use of co-knit, stiches, or alternate loops, the force needed to stretch the textile increased, whereby the co-knit with conductive stitch required the highest force. While for all knitted textiles, the electrical resistance decreased with stretching (loading) and increased upon relaxation (unloading), we did not find any direct relationship between their strain sensing behavior and their cyclic stretch-relaxation profile. The textile sensors showed reproducible response (after up to about 50 cycles) when cyclically stretched and relaxed at 100% applied strain for 500 cycles (Fig. 4).

**Fig. 5:**
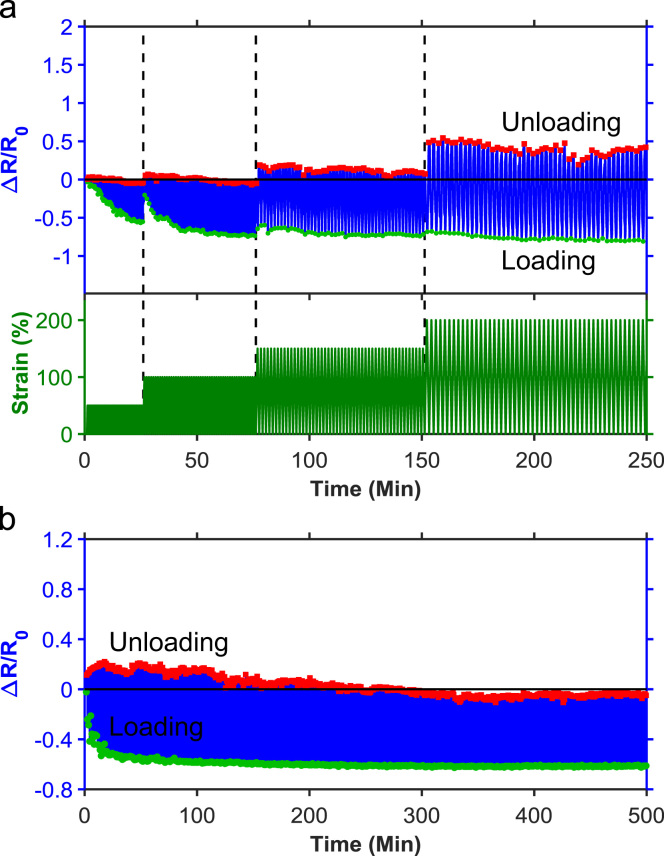
Strain sensing property of the plain-knit fabric using electromechanical tests (a) at different applied strain magnitudes and (b) long-term cycling at 100% stretching.

**Fig. 6:**
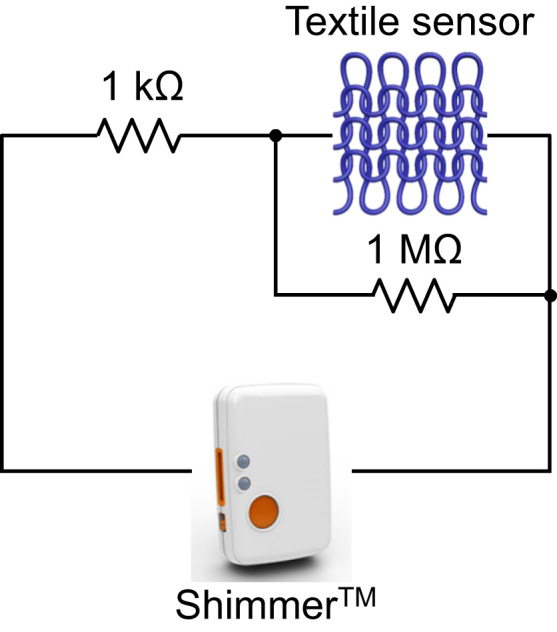
Connection of the knitted sensor prototype to the Shimmer^TM^ wireless transmitter through a voltage divider circuit.

We used a conventional circular knitting machine and made knitted fabrics consisting of plain-knit patches of PU/PEDOT:PSS multifilaments (see Ref. [1] for details of the knitted fabric). The electromechanical testing on the fabric (Fig. 5) at various cyclic strains showed a similar strain sensing behavior to the plain-knit textile structure (see Ref. [1] for the data). The fabric also showed a stable sensing response during 500 cycles of stretch-relaxation test at 100% applied strain. The knitted fabric and textile prototypes could be worn directly on various body parts such as knee, elbow, and finger without the need for an additional supporting substrate, frame, or garment. By interfacing the knitted prototypes with a commercial wireless transmitter (Shimmer^TM^), we were able to monitor the sensing response of the knitted prototypes on a personal computer using the Bluetooth® connection. Fig. 6 shows the connection of Shimmer^TM^ with the knitted prototypes. The knitted sensors responded to diverse bending deformations of knee, elbow, and finger including very fast actions such as kicking (see Videos 2–4 on wireless strain sensing of the knitted prototypes worn on knee, elbow, and finger). In all cases, the voltage signal of the sensor decreased when the limb was bent and then increased upon straightening similar to the wired connection (see Ref. [1] for the wireless strain sensing behaviors of the knitted prototypes).

## 2 Experimental design, materials and methods

Details of materials, experimental methods (preparation of PU/PEDOT:PSS spinning formulations, scaled-up wet-spinning of multifilaments, and knitting multifilaments into textiles and fabrics), and testing procedures (scanning electron microscopy and mechanical and strain sensing measurements) used to generate the data reported here, are described in Ref. [1].

The wireless strain sensing experiments were carried out by connecting the knitted prototypes to the analog expansion (AnEx) board of the Shimmer^TM^ sensor. The AnEx board allows the Shimmer^TM^ platform to connect to a third party analog devices (the textile strain sensor in this case) [4]. A simple voltage divider circuit consisting of two resistors (1 MΩ and 1 kΩ) was used to connect the knitted prototypes to Shimmer^TM^ (Fig. 6) and the signal was transmitted to and was recorded in a personal computer *via* Bluetooth® connection.

## References

[1] Seyedin S., Moradi S., Singh C., Razal J.M. (2018). Continuous production of stretchable conductive multifilaments in kilometer scale enables facile knitting of wearable strain sensing textiles. Appl. Mater. Today.

[2] Seyedin M.Z., Razal J.M., Innis P.C., Wallace G.G. (2014). Strain-responsive polyurethane/PEDOT:pss elastomeric composite fibers with high electrical conductivity. Adv. Funct. Mater..

[3] Seyedin S., Razal J.M., Innis P.C., Jeiranikhameneh A., Beirne S., Wallace G.G. (2015). Knitted strain sensor textiles of highly conductive all-polymeric fibers. ACS Appl. Mater. Interfaces.

[4] Burns A., Greene B.R., McGrath M.J., O’Shea T.J., Kuris B., Ayer S.M. (2010). SHIMMER^TM^ – a wireless sensor platform for noninvasive biomedical research. IEEE Sens. J..

