# Supplementary material for: Data on kilometer scale production of stretchable conductive multifilaments enables knitting wearable strain sensing textiles
Source: Data Brief. 2018 May 1;18:1765–72. doi: 10.1016/j.dib.2018.04.090 (PMC5998204; doi:10.1016/j.dib.2018.04.090)
Supplement: Supplementary file 1 — Supplementary material [file mmc1.docx]

Conflict of Interest

The authors declare that there is no conflict of interest.
