# Supplementary material for: Data on kilometer scale production of stretchable conductive multifilaments enables knitting wearable strain sensing textiles
Source: Data Brief. 2018 May 1;18:1765–72. doi: 10.1016/j.dib.2018.04.090 (PMC5998204; doi:10.1016/j.dib.2018.04.090)
Supplement: Supplementary file 2 — Supplementary material [file mmc2.docx]

ble S1. Summary of trial conditions to achieve PU/PEDOT:PSS multifilaments

| PU  (mg mL^-1^) | PEDOT:PSS  (mg mL^-1^) | Loading  (%) | Filaments | Solvent | Setup | Coagulation | Washing | Drying | Observation |
| --- | --- | --- | --- | --- | --- | --- | --- | --- | --- |
| 200 | - | 0 | 30 | DMF^1^ | Lab-scale | IPA^2^/Water 50/50 (V/V) | Water | - | Filaments sticking |
| 200 | - | 0 | 30 | DMF | Lab-scale | IPA/Water 50/50 (V/V) | Ethanol/Water 80/20 (V/V) | - | Successful |
| 200 | - | 0 | 30 | DMSO^3^ | Lab-scale | IPA/Water 50/50 (V/V) | Ethanol/Water 80/20 (V/V) | - | Filaments sticking |
| 200 | - | 0 | 30 | DMSO | Lab-scale | IPA/Water  90/10 (V/V) | Ethanol/Water 80/20 (V/V) | - | Successful |
| 200 | 20 | 9 | 30 | DMSO | Lab-scale | IPA/Water  90/10 (V/V) | Ethanol/Water 80/20 (V/V) | - | Failed |
| 200 | 20 | 9 | 30 | DMSO | Lab-scale | IPA/Water  80/20 (V/V) | Ethanol/Water 80/20 (V/V) | - | Filaments sticking |
| 200 | 20 | 9 | 30 | DMSO/DMF 50/50 (V/V) | Lab-scale | IPA/Water  80/20 (V/V) | Ethanol/Water 80/20 (V/V) | - | Successful |
| 200 | 30 | 13 | 30 | DMSO/DMF 50/50 (V/V) | Lab-scale | IPA/Water  80/20 (V/V) | Ethanol/Water 80/20 (V/V) | - | Successful |
| 200 | 20 | 9 | 30 | DMSO/DMF 50/50 (V/V) | Scaled-up | IPA/Water  80/20 (V/V) | Water | - | Wet fibers |
| 200 | 20 | 9 | 30 | DMSO/DMF 50/50 (V/V) | Scaled-up | IPA/Water  80/20 (V/V) | Ethanol | 80 °C | Successful |
| 200 | 20 | 9 | 100 | DMSO/DMF 50/50 (V/V) | Scaled-up | IPA/Water  80/20 (V/V) | Ethanol | 80 °C | Successful |
| 200 | 30 | 13 | 100 | DMSO/DMF 50/50 (V/V) | Scaled-up | IPA/Water  80/20 (V/V) | Ethanol | 80 °C | Successful |
| 200 | 35 | 15 | 100 | DMSO/DMF 50/50 (V/V) | Scaled-up | IPA/Water  80/20 (V/V) | Ethanol | 80 °C | Successful, some filament breakage and spinneret blockage |

^1^Dimethyformamide, ^2^Isopropanol, ^3^Dimethylsulfoxide
